# Tips and Tricks for Successful Percutaneous Cryoablation of Large Renal Cell Carcinomas

**DOI:** 10.3390/tomography8050217

**Published:** 2022-10-15

**Authors:** Islam A. S. Elhelf, Hashim Armashi, Arthur Freedman

**Affiliations:** Interventional Radiology, Department of Radiology & Imaging, Medical College of Georgia, Augusta University, Augusta, GA 30912, USA

**Keywords:** cryoablation, percutanous ablation, RCC, renal cell carcinoma, renal ablation

## Abstract

Percutaneous cryoablation has proved to be safe and effective for the treatment of stage T1a renal cell carcinoma (RCC). Patients with larger-sized RCCs may not be good surgical candidates or may have tumors located in anatomically unfavorable locations, which makes partial nephrectomy more challenging. In this patient population, percutaneous cryoablation can be considered a treatment option, given its less invasive nature when compared to surgery. The ablation of larger-sized RCCs requires careful planning to ensure that the tumor volume is completely covered within the ablation zone, while minimizing the risks of non-target injury to the surrounding critical organs. In this article, we share our institutional experience in treating larger-sized RCCs (> 4 cm) using percutaneous cryoablation alone. We discuss strategies to maximize the volume of the ablation zone through the precise placement of the probes. We also shed light on different techniques to protect the surrounding structures during cryoablation.

## 1. Introduction

Thermal ablation is one of the main modalities for the treatment of renal cell carcinoma (RCC). Among the different ablation techniques, cryoablation is widely used with a high safety profile, as well as favorable cure rates [1,2,3]. Favorable outcomes with thermal ablation are usually achieved when the tumor, in addition to at least 5 mm of the surrounding margin, is included within the ablation zone [1,4]. This can easily be achieved in a stage T1a RCC, where the tumor size is ≤4 cm and is limited to the kidney [5].

However, there is a growing need in clinical practice for the percutaneous treatment of larger-sized RCCs. Many patients with larger-sized RCCs are poor surgical candidates. In addition, larger-sized RCCs usually have endophytic/central components or can be located in unfavorable anatomic locations. These characteristics make partial nephrectomy more challenging [6]. Different interventional radiology (IR) techniques have been adopted to treat larger RCCs, including combining trans-arterial embolization with thermal ablation to increase the size of the ablation zone [7,8]. Several publications have shown the feasibility of using cryoablation for the treatment of larger RCCs [1,2,4]. In this article, we share our institutional experience in treating RCCs of >4 cm using percutaneous cryoablation alone.

## 2. Technique

In our institution, we prefer to use general anesthesia (GA) for the ablation of large RCCs. We use the *ICEfx ™* system (Boston Scientific, MA, USA); however, the concepts discussed herein apply to any cryoablation platform in clinical practice.

**Figure 1:**
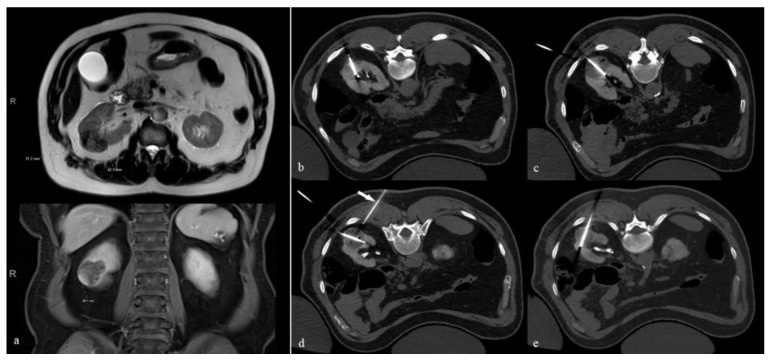
Cryoablation of a right renal RCC. (**a**) MRI scan shows a right renal partly exophytic mass, measuring 4.2 × 3.1 × 4.5 cm along the axial and craniocaudal dimensions, respectively. (**b**–**e**) Four cryoablation probes are carefully aligned along the longitudinal axis of the mass from cranial to caudal. In our experience, the orientation of the probe shown in figure (**e**) is critical as it allows the ice ball to ablate the most central part of the mass, which is a common site for recurrence. The arrow in (**d**) points to a 21 G needle used for CO_2_ dissection, as shown in Figure 2.

Generally, better outcomes can be achieved by placing cryoprobes parallel to the longitudinal axis of the lesion. This is particularly important when using cryoprobes with larger ablation zones, such as the IceFORCE ^®^ 2.1 CX (Galil Medical Inc., MN, USA), which usually produces an ice ball that is greater in length than width. However, the non-uniform shape of these tumors can make probe placement along the longitudinal axis technically challenging. In our experience, the most challenging portions to ablate are the central and medial aspects of the tumor. These are also frequent sites of recurrence [9]. In this case, we recommend placing at least one probe along the longitudinal access of the lesion in the craniocaudal center and toward the medial aspect of the tumor (Figure 1).

Multiple cryoprobes, with 3–5 probes on average, are usually needed to treat large RCCs. To allow for the appropriate coalescence of the separate ice balls, we recommend a spacing of 1.5–2 cm between the probes. In our practice, we typically start by targeting the cranial and then the caudal ends of the tumor. We then place at least one probe along the longitudinal axis at the central/medial aspect of the lesion. We then fill in the gaps between the three initial probes using 1–2 additional probes. An effort is made to avoid traversing the back musculature, if possible. Care is also taken to minimize crossing the normal renal parenchyma as much as possible, although exceptions can be made to ensure appropriate coverage of the target lesion. Careful planning is essential to avoid non-target freezing of intercostal nerves, the genitofemoral nerve, and adjacent bowel loops.

In our practice, we obtain core biopsies with an 18G needle after placing the probes, still prior to initiating ablation. Following the biopsy, we recommend a CT scan to check the probes’ position and allow for final adjustments, prior to starting cryoablation.

Our ablation protocol varies, depending on the size and location of the mass. Most commonly, we perform an 8–10 minute freeze cycle, followed by 6–10 minutes of active thaw. This cycle is repeated for a total of two cycles. We recommend a CT scan five minutes after the start of the freezing cycle, in addition to a scan at the end of the freezing cycle.

It is important to ensure that the surrounding structures are protected prior to starting ablation cycles. Larger lesions, particularly those in the medial lower pole, usually have an endophytic component close to the renal pelvis and/or ureter. For these lesions, we recommend the pre-procedural retrograde urologic insertion of a ureteric stent for pyeloperfusion. We start pyeloperfusion, using warm saline at a rate of 100 cc/hr, ten minutes prior to ablation, and continue this throughout the procedure.

**Figure 2:**
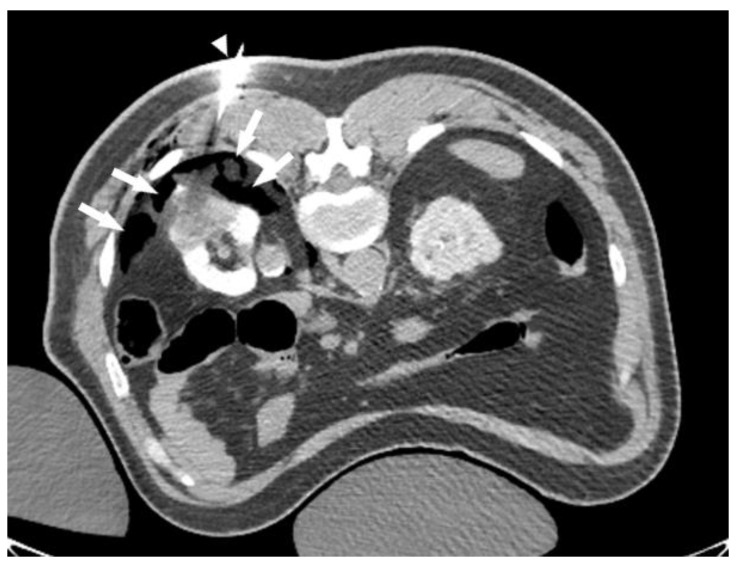
CO_2_ dissection. CT scan of the same patient in Figure 1 during ablation. CO_2_ has been injected through a 21 g needle (white triangle). Notice that CO_2_ (arrows) accumulates in the most posterior, non-dependent, fat planes, creating an insulating plane between the kidney and intercostal nerves.

Another safety consideration is the proximity of exophytic lesions to the surrounding organs, including the genitofemoral nerve, along the psoas muscle, the intercostal nerves, and the adjacent viscera, including the colon, liver, and spleen. We almost always use dissection techniques (using 20–21 G needles) to protect these structures. We recommend using CO_2_ dissection to protect the posterior structures (Figure 2). For dependent structures, such as the colon and liver, we may use normal saline to hydro-dissect these structures away from the ablation zone.

In our practice, we perform tract cauterization prior to removing the probes, to minimize bleeding risk. We also perform a non-contrast abdominal CT scan five minutes after probe removal to check for perinephric hematomas or any other non-target thermal ablation. 

## 3. Discussion

The ablation of RCCs > 4 cm can be technically challenging; however, it is very rewarding, especially in non-surgically fit patients. There are two main factors that have to be considered when ablating large RCCs. The first consideration is safety. The ablation of large RCCs requires the formation of a large ice ball, which may carry a risk of accidental injury to the surrounding organs. A review of the pre-procedure CT/MRI scan is essential to predict potential threats to nearby organs and plan accordingly. In our experience, dissection techniques, using CO_2_ or normal saline, are very effective in this context. We prefer CO_2_ dissection to protect the posterior structures, such as intercostal nerves. When patients are in a prone position, CO_2_ accumulates in the non-dependent fat planes, which provide excellent protection for the posterior structures (Figure 2). For RCCs close to the collecting system, pyeloperfusion using the protocol described has been very helpful to minimize trauma to the ureter and renal pelvis. Patients may develop mild self-limited hematuria following ablation; however, we did not experience more serious complications, such as urine leaks or ureteric strictures. In our experience, cryoablation is safe when performed adjacent to the renal vasculature, but careful attention is needed during the advancement of the probes to avoid direct injury to the hilar vessels. In general, using multiple probes increases the risk of bleeding, given the hypervascular nature of most RCCs. In addition, careful spacing and alignment of the probes are needed to ensure uniform ice ball formation, especially when ablation is close to the renal hilum. Of note, post-procedural bleeding and perinephric hematoma are common with cryoablation. Most of these hematomas are self-limited and do not require further intervention. If there is clinical concern, we admit patients overnight for the observation and monitoring of hemoglobin and hematocrit values every 8 hours. If there is concern about active bleeding, we have a low threshold for performing a renal angiogram and embolization of the bleeding source.

It is noteworthy to mention that different institutions have different policies regarding the biopsy of renal mass lesions. While some institutions perform the biopsy in a separate session prior to ablation, in our institution, we biopsy the lesion during the same session prior to starting ablation. We do not wait for the biopsy results to begin ablation since the decision to ablate is based on suspicious imaging findings. In the majority of cases, histopathology confirms malignancy, which is mostly clear cell carcinoma. Of note, cryoablation platforms may have the option to fix or “stick” the cryoprobes in place by virtue of creating a small size ice ball at the tip of the probe. We recommend not to “stick” the probes prior to obtaining core biopsies. In our experience, ice forming at the probe tip can harden the tissue and make sampling difficult.

The second important factor to consider is efficacy. The ablation of large RCCs requires appropriate planning to ensure complete coverage of the tumor and minimize the chances of leaving residual viable tissue. For this reason, we prefer to perform cryoablation under a general anesthetic (GA). GA allows for appropriate breath-holding during the procedure. This is extremely useful for larger tumors, which necessitate multiple cryoprobes being positioned and spaced with precision. We always place our probes along the longitudinal axis of the lesion to allow for the inclusion of most of the tumor within the growing size of the ice ball.

As mentioned, an average of 3–5 probes are needed for the ablation of large RCCs. Using multiple probes with such tight spacing can be technically confusing. Thus, we recommend attempting to place the probes along different trajectories to easily identify the individual probes. Ideally, the ice balls formed by individual cryoprobes should coalesce into a uniformly large-sized ice ball that covers the whole volume of the tumor. However, tissue characteristics, including perfusion and necrosis, may interfere with this process. As such, it is important to check on the growth of the ice ball with an intra-procedural CT scan to ensure adequate coverage of the target mass and confirm that the ablation does not extend beyond the intended ablation volume. In our experience, the size of the ice ball formed by the second freezing cycle is slightly larger than in the first cycle. Judging the adequacy of ablation should thus be performed at the end of the second freezing cycle.

In conclusion, cryoablation can be used to ablate RCCs of > 4 cm. The large size of these lesions requires specific strategies to maximize the size of the ablation zone while minimizing the chances of collateral damage to the surrounding structures. Familiarity with cryoablation devices, in addition to operator experience, is essential to achieve effective and safe ablation.

## Data Availability

Not applicable.
